# Unravelling the trophic interaction between a parasitic barnacle (*Anelasma squalicola*) and its host Southern lanternshark (*Etmopterus granulosus*) using stable isotopes

**DOI:** 10.1017/S0031182022001299

**Published:** 2022-12

**Authors:** A. J. M. Sabadel, P. Cresson, B. Finucci, J. Bennett

**Affiliations:** 1 Department of Zoology, University of Otago, PO Box 56, Dunedin 9045, New Zealand; 2 IFREMER, Channel and North Sea Fisheries Research Unit, 150 Quai Gambetta, BP 699, 62 321 Boulogne sur Mer, France; 3 National Institute of Water and Atmospheric Research (NIWA), 301 Evans Bay Parade, Hataitai, Wellington 6021, New Zealand

**Keywords:** Deepwater, food web, host–parasite, New Zealand, nitrogen, parasite, shark, stable isotopes, trophic position

## Abstract

The parasitic barnacle, *Anelasma squalicola*, is a rare and evolutionary fascinating organism. Unlike most other filter-feeding barnacles, *A. squalicola* has evolved the capability to uptake nutrient from its host, exclusively parasitizing deepwater sharks of the families Etmopteridae and Pentanchidae. The physiological mechanisms involved in the uptake of nutrients from its host are not yet known. Using stable isotopes and elemental compositions, we followed the fate of nitrogen, carbon and sulphur through various tissues of *A. squalicola* and its host, the Southern lanternshark *Etmopterus granulosus*, to better understand the trophic relationship between parasite and host. Like most marine parasites, *A. squalicola* is lipid-rich and clear differences were found in the stable isotope ratios between barnacle organs. It is evident that the deployment of a system of ‘rootlets’, which merge with host tissues, allows *A. squalicola* to draw nutrients from its host. Through this system, proteins are then rerouted to the exterior structural tissues of *A. squalicola* while lipids are used for maintenance and egg synthesis. The nutrient requirement of *A. squalicola* was found to change from protein-rich to lipid-rich between its early development stage and its definitive size.

## Graphical abstract

**Figure:**
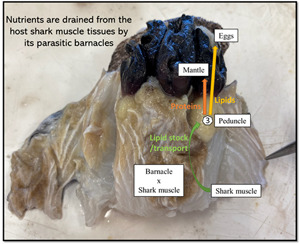

## Introduction

Evolutionary transitions to parasitism are very common in nature. Weinstein and Kuris (2016) estimated that parasitism has independently evolved over 200 times on the tree of life. One unique and fascinating transition involves the barnacle *Anelasma squalicola* Darwin, 1852 (Family Zevina, 1980; https://www.marinespecies.org/aphia.php?p=taxdetails&id=106054), which infects deepwater sharks of the Etmopteridae and Pentanchidae families (Rees *et al*., 2019). This barnacle is known to have a wide host and geographic distribution (Newman and Foster, 1987). Although *A. squalicola* is relatively uncommon in nature (Rees *et al*., 2019), it can reach prevalence as high as 7% [calculated from Yano and Musick (2000)]. Sharks can host between 1 and 4 barnacles embedded in tissues throughout the body, including the head, mouth, fins, abdomen, claspers and cloaca (Yano and Musick, 2000). *Anelasma squalicola* is suspected to have detrimental impacts on the health of their host, and the site of attachment is important for assessing the impact to host from damages caused by the parasite, e.g. when *A. squalicola* attaches on tissue around the gonads, they can retard the development of reproductive organs and thus, impact fecundity (Hickling, 1963; Yano and Musick, 2000).

Unsurprisingly, *A. squalicola*'s life cycle is not well-documented. Frost (1928) first reported a free-living nauplius stage, of which he stated that the morphology of *A. squalicola* strongly contrasts the morphology of filter-feeding barnacle nauplius. Presumably, a free-living cypris stage exists, and then larvae somehow adhere themselves to their shark hosts and develop into their adult forms. Once attached, *A. squalicola* burrows into the flesh of its host by deploying a system of rootlets that will also be used to acquire nutrients from the host (Hickling, 1963; Long and Waggoner, 1993). Once settled, the barnacle can grow to maturation quite rapidly (Ommundsen *et al*., 2016).

Only recently was *A. squalicola* confirmed as a true parasite, primarily because parasitism has only evolved a few times in the history of barnacle species (Cirripedia: Thoracica) (Ommundsen *et al*., 2016). Other vertebrates, such as whales, sea snakes and turtles, are commonly infected with suspension feeding phoresy barnacles. However, of the over 1000 species of stalked and acorn cirripeds, *A. squalicola* is the only non-epibiotic suspension feeder that feeds off the tissue of a vertebrate host (Ommundsen *et al*., 2016). The supporting evidence for determining that *A. squalicola* has a parasitic feeding mode was that their alimentary tracts were void of food items, with their mouth parts reduced and appeared functionally redundant. This hypothesis was also confirmed through stable isotope analyses conducted on barnacles' mantle tissues and compared to their filter-feeding organs (Ommundsen *et al*., 2016). Results indicated that compared to filter-feeding barnacles, *A. squalicola* had different stable isotope values, confirming the impossibility for *A. squalicola* to be feeding on surrounding particulate organic matter, and thus, only leaving the option of a parasitic lifestyle (Ommundsen *et al*., 2016). However, these results could have been tainted by the isotopic gradient usually observed between onshore shallow settings, where the filter-feeding barnacles were collected, and offshore deepwater settings, where the host sharks were caught. Furthermore, stable isotope analyses on the host muscle tissues were not conclusive as the ‘predator–prey’ framework used in stable isotope ecology does not suit parasite–host interactions (Sabadel *et al*., 2019; Thieltges *et al*., 2019; Riekenberg *et al*., 2021*a*).

Stable isotope ratios of carbon and nitrogen, and more recently of sulphur (*δ*^13^C, *δ*^15^N and *δ*^34^S, respectively) have been widely used in ecology (Connolly *et al*., 2004; Fry, 2006). They represent a powerful tool to understand the trophic relationship between a consumer and its food source. Indeed, carbon isotopic ratios do not vary considerably with each trophic level (~1‰), allowing the use of this element as a tracer of organic matter source (Post, 2002; Fry, 2006). Moreover, the relative depletion in *δ*^13^C values is correlated with the presence of lipids, an important food resource for marine parasites (Sabadel and MacLeod, 2022). Similarly, *δ*^34^S values, mainly represented by 2 amino acids, cysteine and methionine, in organic tissues show little change with trophic transfer (Peterson *et al*., 1985; Krouse, 1991). On the contrary, nitrogen is gradually enriched through trophic transfer (~3.4‰), leading to high *δ*^15^N values at high trophic levels (Post, 2002; Layman *et al*., 2012), and allows for inferences of trophic position for a given species.

The stable isotope framework has been fine-tuned over decades to study predator–prey interactions; and more recently, this technique has also been increasingly utilized to help understand the trophic ecology of parasites (Sabadel *et al*., 2016, 2019; Kanaya *et al*., 2019; Sures *et al*., 2019; Thieltges *et al*., 2019; Kamiya *et al*., 2020; Sánchez Barranco *et al*., 2020; Taccardi *et al*., 2020). The ability to select macromolecules from their host (while predators consume their whole prey) may explain the odd isotopic fractionation factors usually reported for parasites and is consistent with the hypothesis of a functional optimization of parasites (Gilbert *et al*., 2020; Riekenberg *et al*., 2021*a*). These recent findings call for more research into the application of stable isotope in parasitology.

The unique evolutionary parasitic lifestyle of *A. squalicola* provides an ideal opportunity to use stable isotopes to understand the physiological mechanisms behind its feeding behaviour. Here, building on Ommundsen *et al*.'s (2016) work, we investigate the relationship between *A. squalicola* and its host, a deepwater Southern lanternshark (or Baxter's dogfish) *Etmopterus granulosus* (Günther, 1880) using stable isotopes and elemental composition of carbon, nitrogen and, for the first time, sulphur, of various parasite and host tissues. We hypothesize that *A. squalicola* depletes its host of lipids, using them as a source of energy to support itself and the next-generation parasitic barnacles. This study provides a pertinent example of the functional transformation associated with the evolution from a free-living filter-feeding life to a parasitic one.

## Materials and methods

### Collection of specimens

**Fig. 1.**
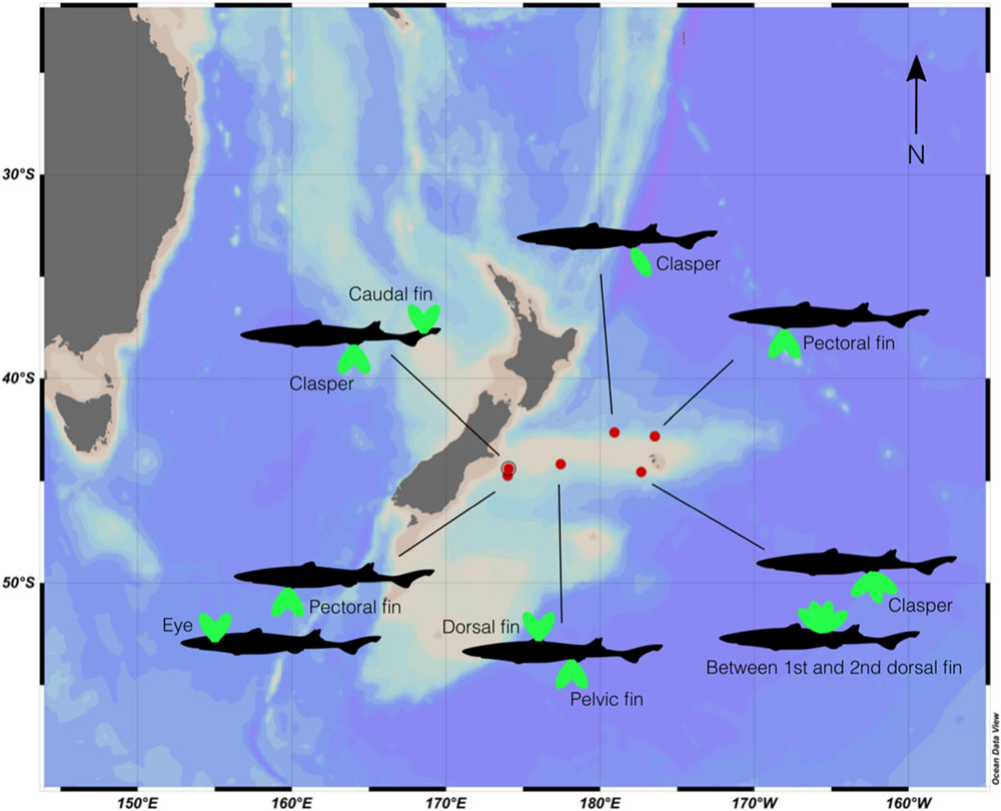
Map depicting the locations where *Etmopterus granulosus* infected with *Anelasma squalicola* were collected on Chatham Rise, New Zealand in January 2022. The number of parasitic barnacles and their site of infection on each host shark are indicated by the green ovals.

Specimens (host and parasite) were obtained during a fisheries independent research trawl survey conducted by the National Institute of Water and Atmospheric Research (NIWA), on board RV *Tangaroa* on Chatham Rise in January 2022 (TAN2201). Trawl surveys were stratified-random with resulting sampling strata defined by location and depth, and fishing occurred on trawlable fishing grounds. Sharks were measured for total length (TL, cm) and visually inspected for signs of parasite infections. Sharks confirmed to have barnacle infections were kept whole, frozen at sea and brought back to the laboratory for analyses. In total, 8 sharks were obtained for this study, representing 22 parasitic barnacles (Fig. 1). Specimens were obtained from depths between 707 and 1261 m depth.

### Shark and barnacle dissections

**Fig. 2.**
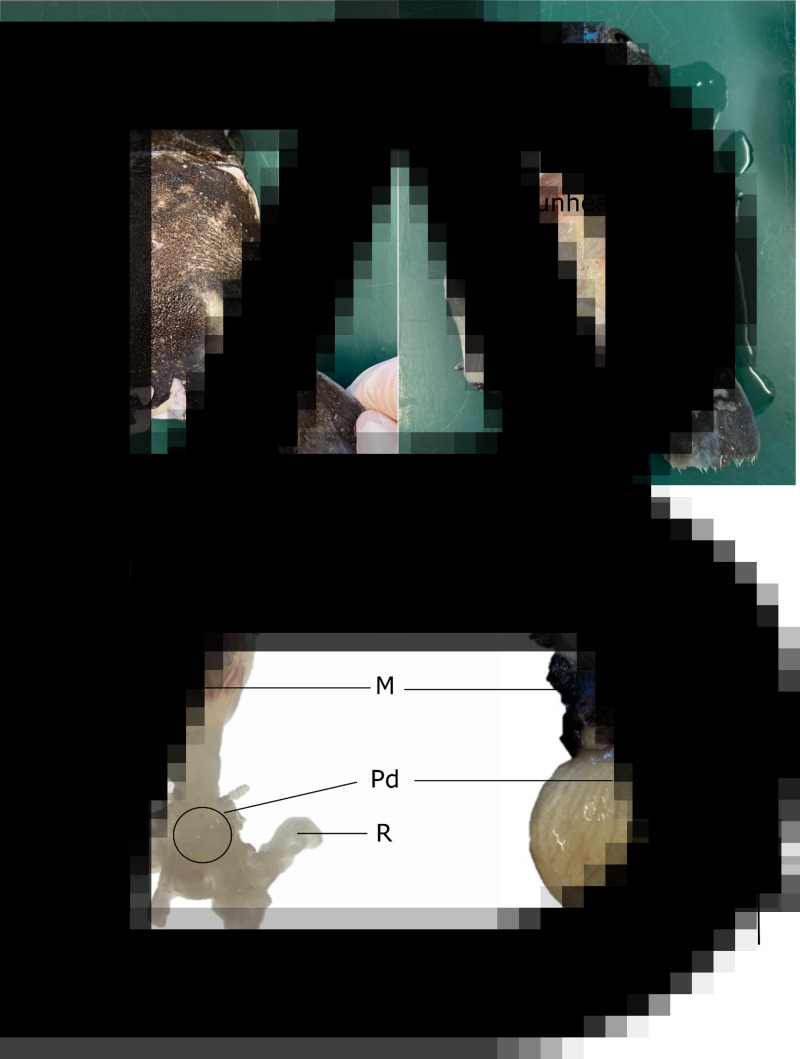
*Anelasma squalicola in situ* on *Etmopterus granulosus*. (A) Pre-dissection photograph of *A. squalicola* infecting *E. granulosus* (left) and partially dissected *A. squalicola* showing ‘unhealthy’ host tissue infested with *A. squalicola* rootlets, Pd, and healthy host tissue (H) (right). (B) Two parasitic barnacles (varying in size) illustrating tissues taken for stable isotope analyses. These include mouth, cirri and penis (MCP), eggs (Egg), mantle (M), peduncle (Pd) and rootlets (R). Not represented is the inner mantle, a soft tissue found within the mantle. Scale bars represent 1 cm.

In the laboratory, sharks were defrosted and their barnacles and approximately 2–3 cm of surrounding host tissue were dissected for stable isotope analysis. A total of 10 infection sites were identified, with 2 of the 8 sharks infected in 2 separate locations. Each site contained either 1 (*n* = 1 site), 2 (*n* = 7 sites), 3 (*n* = 1 site) or 4 (*n* = 1 site) barnacles embedded together. For the host shark, ‘healthy’ muscle tissue was collected, close to each barnacle's sites, but beyond the reach of the rootlets (*n* = 22) (Fig. 2A). For 2 of the sharks, we also collected tissues that were clearly impacted by the presence of the barnacle. This tissue was labelled as ‘unhealthy’ (Fig. 2A). For each barnacle, we isolated the following tissues: mantle (*n* = 22), mouth + cirri + penis (MCP, *n* = 21), rootlets (*n* = 22), peduncle (*n* = 22) and eggs (*n* = 12) (Fig. 2B). All tissues were placed in individual Eppendorf tubes, and dried in an oven at 60°C for at least 48 h. We used the dried weight of the entire mantle as a proxy for barnacle size/age and categorized all individuals in one of the following size classes: small < 50 mg, medium 50 mg < weight < 100 mg and large > 100 mg.

### Bulk stable isotope measurements

Stable isotope ratios of shark and barnacle tissues were measured at the Isotrace Lab in Dunedin, New Zealand. For each sample, approximately 0.8 mg of dried material was packed into a tin capsule and folded prior to stable isotope measurements. None of the samples were lipid-extracted so that the lipids impact on the *δ*^13^C values was captured, as these were expected to be an important food resource to parasites. Values of *δ*^15^N, *δ*^13^C and *δ*^34^S, along with the elemental compositions of carbon, nitrogen and sulphur, were measured on an EA Isolink CNSOH coupled with a Delta *vs* V Advantage Isotopic Ratio Mass Spectrometer (Thermo Fisher, Waltham, MA, USA). The stable isotope values are reported as: *δX* = [(*R*_sample_/*R*_standard_) − 1] × 1000 where *X* is the element ^13^C, ^15^N or ^34^S, and *R* is the corresponding isotope ratio ^13^C/^12^C, ^15^N/^14^N or ^34^S/^32^S, respectively. The standards used to calibrate the *δ* values were Vienna Peedee Belemnite (VPDB) for carbon, atmospheric N_2_ for nitrogen or Canyon Diablo troilite (CDT) for sulphur. The samples were standardized to international isotope reference materials G01, a mix of USGS40 and IAEA-S1 (*δ*^15^N = −4.52‰, *δ*^13^C = −26.39‰ and *δ*^34^S = −0.30‰) and G02, a mix of USGS41 and IAEA-S2 (*δ*^15^N = 47.55‰, *δ*^13^C = 36.55‰ and *δ*^34^S = 22.62‰). The quality control was conducted by applying an in-house laboratory control material, Keratin Internal Standard (*δ*^15^N = 8.91‰, *δ*^13^C = −21.14‰ and *δ*^34^S = 13.08‰). Instrument precision was 0.05‰ for *δ*^15^N values, 0.07‰ for *δ*^13^C and 0.60‰ for *δ*^34^S.

### The specific case of the barnacles in the eye

**Fig. 3.**
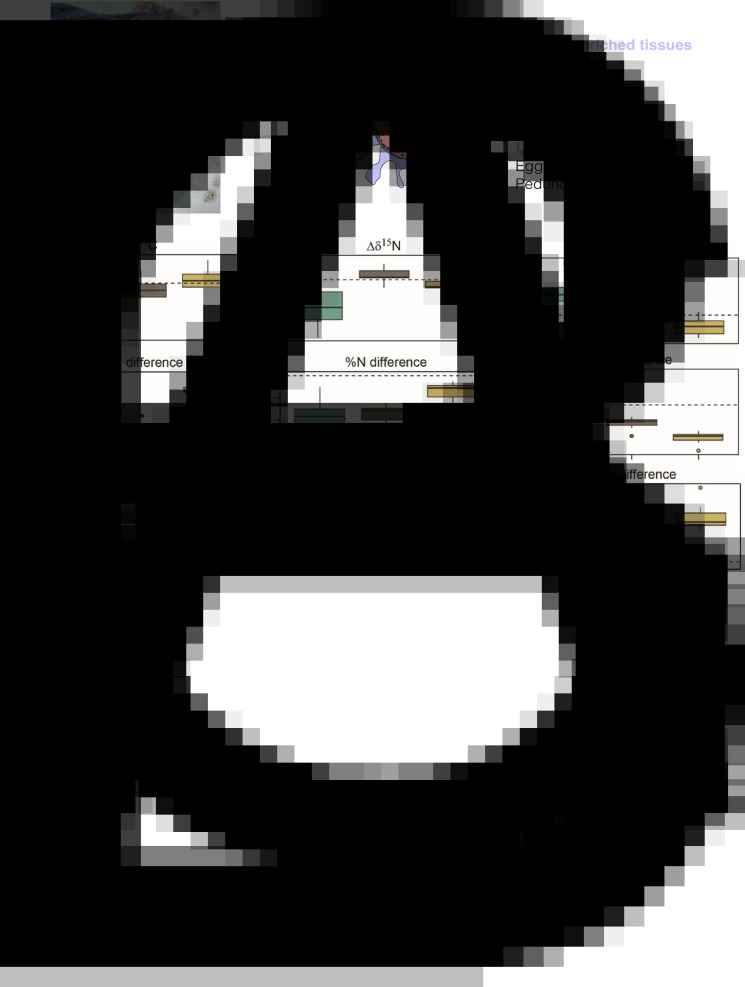
(A) *Anelasma squalicola in situ* of the right eye of *E. granulosus* whereby rootlets appear to have penetrated host cartilage for access to host muscle in the centre of the shark head. (B) Visual characterization of *A. squalicola* identified as either protein-rich (purple) or lipid-rich (pink) tissues. (C) Stable isotope values and elemental compositions differences between parasite and host tissues. The difference between all barnacle ‘protein tissues’ (mean of all barnacles except individuals on shark no. 11 and their matching shark ‘healthy’ muscle tissues; green); the difference between shark no. 11′s barnacle ‘protein tissues’ and the eye tissue of the shark (grey); and the difference between shark no. 11 barnacle's ‘protein tissues’ and the ‘healthy’ shark muscle tissue (yellow).

One shark (shark no. 11) had 2 small barnacles embedded in its right eye. The barnacles appeared to embed in the vitreous of the eye and penetrate the cartilage behind with their rootlets to access muscle behind the cartilage (Fig. 3A). We took this opportunity to investigate if *A. squalicola* fed on the tissues at the site of infection (i.e. the eye), or beyond site of infection where the rootlets are located (i.e. the muscle behind the eye cartilage). We used the ‘protein tissues’ (average values of the mantle, the rootlets, the inner mantle and the MCP tissues; Fig. 3B) of all barnacles from this study (except those of shark no. 11) and estimated the differences (Δ) in stable isotopes values and elemental composition between barnacles and shark ‘healthy’ muscle tissues (Fig. 3C), e.g. Δ^15^N_Parasite–Host_ (‘healthy’ muscle) = *δ*^15^N_Parasite_ (‘protein tissues’) – *δ*^15^N_Host_ (‘healthy’ muscle). Differences were calculated for all barnacle–shark pairs excluding shark no. 11, then compared to the barnacles from shark no. 11 *vs* the host eye tissues and *vs* the host muscle behind the eye cartilage.

### Statistical analyses and parameters

The elemental C/N ratio is commonly used as a proxy for lipid-rich *vs* protein-rich tissues, with a high ratio indicating the former and a low ratio the latter. Differences in isotopic and elemental content were compared by analysis of variance (ANOVA), followed by Tukey post-hoc tests. Correlation between stable isotope values, elemental compositions and biotic and abiotic parameters (shark length, latitude and longitude) was estimated using Pearson's correlation coefficient. All statistical analyses were run using R version 4.1.2 and the packages *multcomp* and *PeformanceAnalytics* (Hothorn *et al*., 2008; Peterson *et al.*, 2020; R Core Team, 2020).

## Results

### Stable isotope values and elemental compositions of the host shark

Of the 439 *E. granulosus* sampled during the TAN2201 voyage, 18 were found to be infected with *A. squalicola* (4% infection prevalence). Eight of these sharks were investigated in this study, covering 6 locations on the Chatham Rise, New Zealand (Fig. 1). Of these sampled sharks, there were 2 females and 6 males, measuring between 38 and 71 cm TL.

The ‘healthy’ muscle tissues of shark hosts had *δ*^15^N values ranging from 9.6 to 14.0‰ (avg. 12.0 ± 1.3‰), *δ*^13^C values from −19.6 to −17.6 ‰ (avg. −18.7 ± 0.8‰) and *δ*^34^S from 19.6 to 21.2‰ (avg. 20.4 ± 0.8‰) (Tables 1 and Sd1). Further, *δ*^13^C values of host ‘healthy’ muscle tissues were significantly and positively correlated with latitude (Pearson's *ρ* = 0.88, *P* ≪ 0.001) and longitude (*ρ* = 0.90, *P* ≪ 0.001) (Supplement Fig. S1), while *δ*^15^N and *δ*^34^S values of the same tissue only correlated with longitude: positively (*ρ* = 0.81, *P* < 0.001) and negatively (*ρ* = −0.82, *P* < 0.001), respectively. We compared stable isotope values of the ‘healthy’ and the ‘unhealthy’ shark muscle and found that the ‘unhealthy’ shark tissues exhibited lower *δ*^15^N and *δ*^13^C, but slightly higher *δ*^34^S values (Table S1). Additionally, ‘unhealthy’ tissues on average contained less nitrogen and slightly more carbon, thus increasing the C/N ratio, which is usually indicative of lipid-rich tissues. The percentage of sulphur was equivalent between ‘healthy’ and ‘unhealthy’ tissues (Table S1).

**Table 1.** Average stable isotope values of N, C and S, along with elemental compositions and C/N ratios of host shark *Etmopterus granulosus* and their parasitic barnacles *Anelasma squalicola*, collected from the Chatham Rise, New Zealand

Host shark		*δ*^15^N (‰)	%N	*δ*^13^C (‰)	%C	*δ*^34^S (‰)	%S	C/N
‘Healthy’ muscle (*n* = 10)	Avg.	12.0	15.5	−18.7	42.7	20.4	1.0	2.7
	s.d.	1.3	1.6	0.8	8.3	0.8	0.2	0.4
Eye (*n* = 1)	Avg.	8.9	14.6	−19.2	45.0	20.1	1.1	3.1
‘Unhealthy’ muscle (*n* = 4)	Avg.	11.5	13.5	−19.1	48.7	20.9	0.9	3.6
	s.d.	0.9	1.1	0.5	2.8	0.4	0.4	0.5
Parasitic barnacle								
Peduncle (*n* = 18)	Avg.	11.7	10.2	−19.9	56.3	21.0	0.7	6.6
	s.d.	1.6	3.3	1.7	11.1	1.0	0.3	3.5
*Mantle (n* *=* *18)*	*Avg.*	*10*.*8*	*12*.*1*	*−19*.*1*	*48*.*7*	*21*.*5*	*0*.*9*	*4*.*1*
	*s.d.*	*2*.*7*	*1*.*8*	*0*.*8*	*6*.*4*	*0*.*8*	*0*.*5*	*0*.*9*
*Inner mantle (n* *=* *18)*	*Avg.*	*10*.*1*	*12*.*1*	*−19*.*2*	*46*.*1*	*21*.*1*	*0*.*7*	*3*.*9*
	*s.d.*	*1*.*6*	*1*.*2*	*0*.*7*	*3*.*4*	*0*.*6*	*0*.*1*	*0*.*6*
*Rootlets (n* *=* *18)*	*Avg.*	*10*.*8*	*12*.*9*	*−19*.*1*	*46*.*2*	*21*.*1*	*0*.*8*	*3*.*6*
	*s.d.*	*1*.*3*	*1*.*3*	*1*.*0*	*4*.*3*	*0*.*6*	*0*.*2*	*0*.*6*
Eggs (*n* = 11)	Avg.	11.0	6.2	−22.1	66.9	19.8	0.4	10.9
	s.d.	1.0	0.5	0.5	2.8	0.8	0.1	1.1
*MCP (n* *=* *19)*	*Avg.*	*10*.*5*	*12*.*4*	*−18*.*8*	*44*.*0*	*21*.*6*	*0*.*9*	*3*.*6*
	*s.d.*	*1*.*4*	*0*.*8*	*0*.*5*	*2*.*4*	*0*.*5*	*0*.*1*	*0*.*4*
***Protein tissues***	***Avg.***	***10***.***6***	***12***.***3***	***−19.0***	***46***.***2***	***21***.***3***	***0***.***8***	***3***.***8***
	***s.d.***	***1.4***	***1***.***1***	***0***.***6***	***2***.***7***	***0***.***5***	***0***.***2***	***0***.***5***

Parasite tissues in *italic* are all part of the ‘protein tissues’ category. Note that S.D.s are not provided for the eye tissue as the measurement was made on one individual only.

Bold and italic represents the average values of all ‘protein’ tissues (Mantle, Inner mantle, Rootlets and MCP).

### Stable isotope values and elemental compositions of parasitic barnacles

The average values for stable isotopes and elemental compositions of *A. squalicola* are reported in Table 1. All data for the various barnacle tissues of individual organisms can be found in the Supplement data (Tables Sd2–9). There were no significant differences (*P* > 0.05) between the mantle, the rootlets, the inner mantle and the MCP for stable isotope values, elemental compositions or C/N ratios (see Pearson's correlations and *post hoc* tests in Table S2). Additionally, the C/N ratios of these 4 tissues are relatively low (avg. 3.6 ± 0.6 to 4.1 ± 0.9) in comparison to the other selected parts of the parasite (C/N_Peduncle_ = 6.6 ± 3.5 and C/N_Eggs_ = 10.9 ± 1.1), thus reflecting protein-rich tissues. As such, the mantle, the rootlets, the inner mantle and the MCP were combined into a ‘protein tissues’ category.

Subsequently, based on the average values of each barnacle tissues (Table 1), the highest *δ*^15^N values were the peduncles (avg. 11.7 ± 1.6‰) and the lowest were the protein tissues (avg. 10.6 ± 1.4‰), although these were not significantly different (Table S2). Conversely, for *δ*^13^C the highest values were the ‘protein tissues’ (avg. −19.0 ± 0.6‰), while the lowest were the eggs (avg. −22.1 ± 0.5‰), where a difference was found between the 2 tissues (Table S2). For *δ*^34^S the highest values were the ‘protein tissues’ (avg. 21.3 ± 0.5‰) and the lowest were the eggs (avg. 19.8 ± 0.8‰).

The barnacles' mantle dried weights were used as a proxy for the parasites size. These mantle weights ranged from 4.85 to 226.67 mg, covering a wide range of sizes. Within the ‘protein tissues’, the size (mantle weight) of *A. squalicola* was strongly and negatively correlated with *δ*^15^N values (*ρ* = −0.75, *P* < 0.001; Fig. S2), *δ*^34^S values (*ρ* = −0.83, *P* < 0.001; Fig. S2) and %S (*ρ* = −0.69, *P* < 0.05; Fig. S2). Further, within the peduncle tissues, the size of *A. squalicola* was negatively correlated with %N (*ρ* = −0.78, *P* < 0.001; Fig. S3) and %S (*ρ* = −0.81, *P* < 0.001; Fig. S3), and positively correlated with %C (*ρ* = 0.83, *P* < 0.001; Fig. S3) and with the C/N ratio (*ρ* = 0.79, *P* < 0.001; Fig. S3). Additionally, the barnacle size was negatively correlated with both the peduncle's *δ*^13^C (*ρ* = −0.81, *P* < 0.05; Fig. S3) and *δ*^34^S values (*ρ* = −0.87, *P* < 0.05; Fig. S3).

The effect of the number of barnacles per infection site (Fig. S4) appeared to show differences in most stable isotope values and elemental compositions for 1 and 3 barnacles in comparison with clusters of 2 and 4 individuals. These observed differences were likely due to a size effect because these barnacles were relatively small compared to the ones that occupied sites as groups of 2 or 4 (Tables Sd2–7 for barnacles' sizes/dried mantle weights).

### The specific case of the barnacles in the eye

For shark no. 11 (i.e. the only shark exhibiting barnacles settled in the eye; Fig. 3A), isotopic or elemental differences between *A. squalicola* and either the eye, or the muscle behind the eye have been plotted in Fig. 3C. The average difference between ‘muscle-embedded’ barnacles (i.e. all other barnacles excluding those of shark no. 11) and the ‘healthy’ muscle tissues of their respective host was used as a reference. This comparison highlighted that the differences between the barnacle from shark no. 11 and the eye were closer to the reference for all carbon and sulphur-related descriptors, including the C/N ratio, but were only holding for %N and not for *δ*^15^N values (Fig. 3C).

## Discussion

We hypothesized that the *A. squalicola* depletes their shark host of lipids and as such, expected the ‘unhealthy’ shark tissue to be lipid-drained by the passive-feeding parasites. However, stable isotope values and elemental compositions indicated that the ‘unhealthy’ shark tissues are in fact, a mixture of barnacle rootlets and shark muscle. Here the rootlets transport nutrients (i.e. majority of lipids and few proteins) from the surrounding ‘healthy’ host muscle tissue to their peduncle, before nutrients are then redistributed to the ‘protein tissues’ and egg stock. This is evidenced by our findings below.

### Unravelling the feeding mechanism of *A. squalicola*

**Fig. 4.**
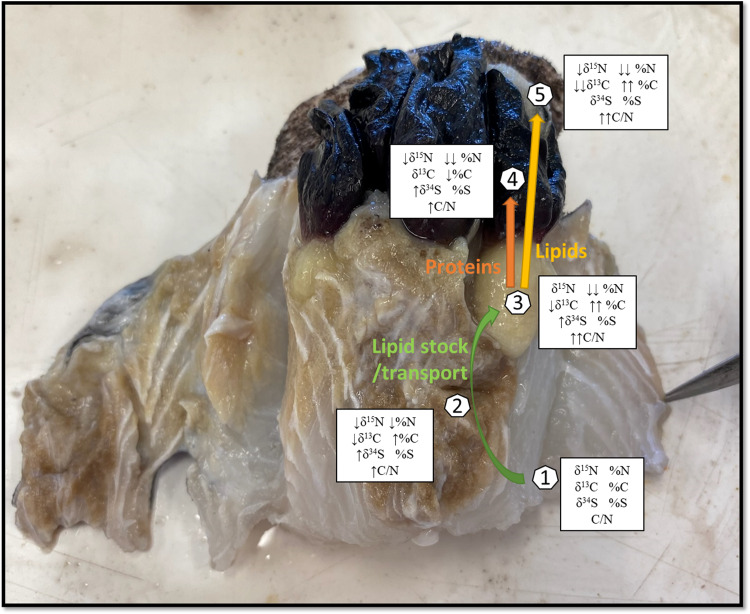
Proposed physiological mechanisms behind parasitic barnacle feeding. (1) ‘Healthy’ shark muscle tissue, (2) ‘unhealthy’ shark tissue, (3) one of the barnacle's peduncle, (4) the same barnacle's protein tissues and (5) its egg stock. Green arrow represents a transfer of lipids and proteins *via* the barnacle's rootlets, orange arrow represents a transfer of proteins for maintenance and yellow arrow represents a transfer of lipids to the next generation.

Higher lipid content than in ‘healthy’ shark muscle tissues were observed in all parasite organs analysed (see %C and C/N ratios in Table 1). This was even more marked in the barnacle's peduncle and egg tissues. In fact, with lipids exhibiting lower *δ*^13^C values than other carbon-containing molecules, the observed depletion gradient along with an increasing carbon content between host and parasite is pointing to a clear path of lipid transport: from ‘healthy’ to ‘unhealthy’ shark muscle tissues, then to the egg stocks *via* the peduncle. In parallel, the ‘protein tissues’, representing the structure of the barnacle, displayed similar *δ*^13^C values and carbon content than that of the ‘unhealthy’ shark muscle tissues and a rather low C/N ratio typical of high protein content. Interestingly, while nitrogen content was statistically different across the various barnacle organs and lower compared to the shark muscle tissues, the *δ*^15^N and *δ*^34^S values, and sulphur content stayed relatively constants. This could be interpreted as a second nutrient pathway from host to parasite, whereby proteins are rerouted to the ‘protein tissues’ after being first absorbed and possibly enzymatically reworked in the ‘unhealthy’ muscle tissues. We illustrated this proposed mechanism of the redistribution of host nutrients to different barnacle organs in Fig. 4.

Further, Ommundsen *et al*. (2016) suggested that the high lipid content of *A. squalicola* may result from the uptake of hosts' interstitial fluid, which is also rich in lipids. If true, and considering our findings, there could be 2 possible scenarios: (1) the intestinal fluid contains depleted host metabolites, and/or (2) the parasite can select the metabolites to incorporate within its own tissues and chooses the most energy efficient (light isotopes-containing ones). However, neither the potential enzymatic reworking nor the fractionation during these metabolite uptakes by the parasite can be perceived by bulk stable isotope analysis, and therefore it is not possible to distinguish between the scenarios and fully characterize the uptake mechanisms. As such, this framework would largely benefit from further investigation into the exact routing of proteins and lipids, e.g. by analysing amino acid or fatty acid compositions of the different tissues. This would allow confirming that protein and lipid demands – and subsequent host-to-parasite nutrient fluxes – do change with growth or reproduction status of the barnacle. In addition, compound-specific stable isotopic analysis (CSIA) of amino acids could also be powerful to ascertain the effect of metabolism on parasite's isotopic ratio and could help tease apart enzymatic activities (Sabadel *et al*., 2019; Riekenberg *et al*., 2021*b*), while CSIA of fatty acid (e.g. polysaturated long-chain fatty acids) could shed light on the routing of lipid from host to parasite (Twining *et al*., 2020). Nevertheless, these results are aligned with other studies looking at ‘absorptive’ parasites such as acanthocephalan (Nachev *et al*., 2017) or cestodes (Power and Klein, 2004; Finn *et al*., 2022), challenging the classic framework of predator–prey relationships (i.e. *δ*^15^N enrichment from prey to predator) (Thieltges *et al*., 2019; Kamiya *et al*., 2020; Sabadel and MacLeod, 2022).

The correlations of each measured variable (stable isotope values and elemental compositions) with barnacle sizes could be indicative of a metabolic shift leading to different nutrient requirements between developing and fully grown organisms. Indeed, it seems that in the early stages of their development, *A. squalicola* requires more protein and less lipids than later in its life, as evidenced by the lower %N, %S, *δ*^13^C, *δ*^34^S and the higher %C, C/N ratio in larger individuals. As such, on one hand, small barnacles require more proteins to grow their structure and less lipids as they are not yet fully reproductively active. Adult barnacles, on the other hand, swap this nutrition style for a lipid-rich diet with relatively less proteins. Lipid dynamics was largely demonstrated as a major driver of host–parasite exchanges, by example for nematodes (Strømnes and Andersen, 2003; Strømnes, 2014; Mille *et al*., 2020). Indeed, egg synthesis in marine environments consists mostly of an accumulation of lipids, which will represent future reserves of energy supporting the early development of newly hatched larvae [e.g. Kolodzey *et al*. (2021)]. The main function of an adult parasite, along with its own maintenance, is to produce and emit eggs. As such, functional simplification must have driven their ability to uptake lipid from their host in order to fuel the eggs' reserves. Results obtained here seem to demonstrate that the important role of lipids in adult barnacles can be generalized to other parasitic groups. However, other parasite tissues such as the ‘protein tissues’ also indicate some reliance on proteins. Further, the high variation in stable isotope values and elemental composition of the peduncle tends to confirm that it is the only feeding organ present, and as such, the nutrients stored in it might change depending on the barnacle's requirements (e.g. depending on its spawning status). The parasite may divert metabolic resources that are required for normal reproductive development in the hosts, which live in deep habitats where energy may be in short supply (Yano and Musick, 2000).

Interestingly, while the *δ*^15^N values from this study matched well the results from Ommundsen *et al*. (2016) for similar tissues (i.e. shark muscle and barnacle mantle), *δ*^13^C values yield the opposite trend: authors found the barnacle to be enriched in *δ*^13^C, which would emphasize the use of protein for the ‘protein tissues’ rather than a combination of protein and lipids. However, it could not be determined whether the barnacle samples had been lipid-extracted prior to stable isotope analysis, as this methodological point is not specified in Ommundsen *et al*. (2016). This would have indeed enriched the *δ*^13^C values of the mantle tissues and represented non-lipid molecules. Extracting lipids from parasites or host tissues prior to stable isotope analyses may blur the pattern of organic matter transfer between host and parasites, as lipids are a key (and sometimes the only) food resource of parasites. Moreover, lipid-extraction protocol has revealed a crucial step in the robust application of stable isotopes in trophic ecology. It is now applied routinely to assess predator–prey interactions, as several calibrations of the seminal protocol proposed by Post *et al*. (2007) allowed the generalization of the method to different conditions [e.g. Kiljunen *et al*. (2006), Logan *et al*. (2008)]. The possible methodological discrepancy observed here seems however to confirm again the need for a similar development of a dedicated theoretical and methodological framework, before being able to apply routinely stable isotopes to host–parasite interactions.

### The specific case of the barnacles in the eye

Most of the barnacles collected for this study were found attached to the sharks' body (e.g. dorsal fin, pectoral fin, tail), or embedded within their claspers. One infection site was in the eye (Fig. 3A). The close resemblance of the differences between the 2 barnacles and the shark eye tissue in the averaged values of all variables – whether stable isotope values or elemental compositions – confirmed that *A. squalicola* likely feeds on the eye rather than on the muscle behind the cartilage of their host's head. Although the small sample size (*n* = 2) precludes from generalization of the pattern observed, this could indicate that the ‘rootlets’, which had pierced through the eye, might not be the mean *via* which *A. squalicola* is feeding, as previously suggested (Hickling, 1963; Long and Waggoner, 1993). Instead, they may only be used for anchoring the barnacle in this instance. In this scenario, the barnacles would be feeding on the shark by mixing the peduncle tissues (i.e. different types of rootlets) with the surrounding host muscle tissues, as indicated by the nature of the ‘unhealthy’ host muscle tissues. This assemblage of barnacle and shark tissues could then become a path for the parasite to channel nutrients, in the form of a fluid in which the peduncle is sitting. Variability of the peduncle stable isotope values and elemental compositions may support the hypothesis of reworking of obtained lipids (e.g. fatty acids) by the peduncle, prior to rerouting them to its eggs stock.

### Other insights

Two *A. squalicola* per infection site was by far the predominant occurrence. Yano and Musick (2000) reported that over 70% of all infection sites had 2 *A. squalicola*. This is supported by our data as 7 of the 10 infections hosted 2 barnacle individuals. In the one case where a single barnacle attached to a shark, the individual was small (mantle dried weight < 50 mg) indicating it was probably an early infection. We also found occurrences of 3 and 4 barnacles per infection site. In the case of the 3 barnacles, while all small, 2 had similar sizes with a third much smaller, possibly indicating their various orders of arrival. For the 4 barnacle infections, all individuals were large in size and were likely parasitizing the shark for some time, as demonstrated by the relatively extensive amount of ‘unhealthy’ shark tissues, compared to other samples (e.g. Fig. 2A infection compared to Fig. 4). There were some differences between individual barnacles within infection sites, but there was no clear positive or negative trend that indicated size – and by extension age – was not the factor influencing these differences. One possibility for this phenomenon could be that as barnacles infect the same site, all the barnacles' rootlets intertwine into 1 common block of barnacle/shark tissue, as indicated by the values of ‘unhealthy’ shark muscle tissue (Tables 1 and S2). This could be advantageous or disadvantageous to individual barnacles depending on their position within the cluster and their access to the nutrients/host metabolites.

The *E. granulosus δ*^13^C values were strongly and positively correlated with latitude and longitude, following the known *δ*^13^C tropical-Antarctic (Graham and Bury, 2019) and the onshore–offshore depletion gradients, respectively. These reflected differences in temperature and the solubility of CO_2_ as observed elsewhere (Goericke and Fry, 1994; Laws *et al*., 1995; Graham *et al*., 2010; Trueman *et al*., 2012) are shown here for Chatham Rise. Stable isotope spatial variations were also marginally observed positively for *δ*^15^N and negatively for *δ*^34^S values across a latitudinal gradient. With stable isotopes representing time-integrated information, this spatial relationship within shark tissues could indicate that these sharks remain resident to a relatively small region, consistent with previous results obtained elsewhere (Bird *et al*., 2018). *Etmopterus granulosus* has a strong affinity to seamount communities (Finucci *et al*., 2018), and although the species has widespread distribution across the Southern Hemisphere (Straube *et al*., 2011), any finer scale population structure is currently unknown. Further, the relatively high *δ*^34^S values obtained for *E. granulosus* seem to indicate offshore pelagic rather than inshore and/or benthic feeding for these sharks (Connolly *et al*., 2004). This finding is however in contradiction with results from visual diet studies (Dunn *et al*., 2013) and warrants further investigation.

Interestingly, the *δ*^13^C gradients observed in the sharks' ‘healthy’ muscle tissues were also detectable within the barnacles but only in the ‘protein tissues’, and across a longitudinal gradient. This lack of gradients could underscore the complex metabolic processes happening within the barnacle, as neither the peduncle nor the egg stock covaried with either latitude or longitude. This finding may be attributed to the parasite's absorptive feeding mode which here again defies the classic predator–prey interactions as the *δ*^13^C values showed little to no fractionation. In addition, organs such as the mouth and cirri (as main parts of the MCP) are structures without function, and may thus have limited metabolic activity since they are no longer used for food capture (Rees *et al*., 2014).

## Conclusion

In this study, we unravel the importance of lipids as a driver of the interaction between the parasitic barnacle *A. squalicola* and its host shark *E. granulosus*. Using stable isotopes, we tracked the flow of N, C and S, and ultimately protein and lipids from host to parasite by passive feeding, i.e. absorption of selected nutrients/host metabolites. This is similar to other passive feeding marine parasites (Sabadel and MacLeod, 2022). *Anelasma squalicola* is a representative of just 1 independent evolutionary transition of the over 200 currently reported in the history of metazoans. Although independent, this particular transition has convergently evolved similar mechanisms to other parasites for which to obtain nutrients. We propose a mechanism whereby the barnacle tissue fusion with the shark muscle tissues, thus creating a mix of parasite and shark tissues that potentially expand in response to increased nutrient demands for parasite, e.g. as the number of barnacle in a cluster increases and with size and/or maturity of an individual parasite. Once the nutrients have reached the peduncle, proteins are rerouted in the ‘protein tissues’, especially in the initial growth spurt of the barnacles, while the lipids are mostly channelled to generate the eggs and secure the next generation. Further research could include fatty acid profiling and both CSIA of fatty acids and amino acids to understand which compounds are absorbed by the barnacle from its host shark. Investigating the relatedness of barnacles that infect the same site would provide great insight into the life cycle of this mysterious parasite.

## Supplementary material

For supplementary material accompanying this paper visit https://doi.org/10.1017/S0031182022001299. click here to view supplementary material
