# Supplementary material for: Unravelling the trophic interaction between a parasitic barnacle (Anelasma squalicola) and its host Southern lanternshark (Etmopterus granulosus) using stable isotopes
Source: Parasitology. 2022 Sep 9;149(14):1976–84. doi: 10.1017/S0031182022001299 (PMC10090636; doi:10.1017/S0031182022001299)
Supplement: Supplementary file 1 [file S0031182022001299sup.zip › S0031182022001299sup002.docx]

**Supplement**

Unravelling the trophic interaction between a parasitic barnacle (*Anelasma squalicola*) and its host the Southern lanternshark (*Etmopterus granulosus*) using stable isotopes

A.J.M. Sabadel^a,*^, P. Cresson^b^ B. Finucci^c^ and J. Bennett^a^

^a^Department of Zoology, University of Otago, PO Box 56, Dunedin 9045, New Zealand
^b^IFREMER, Channel and North Sea Fisheries Research Unit, 150 Quai Gambetta, BP 699, 62 321 Boulogne sur Mer, France.

^c^NIWA, 301 Evans Bay Parade, Hataitai 6021, Wellington, New Zealand

*Corresponding author: amandine.sabadel@otago.ac.nz

**Figures**


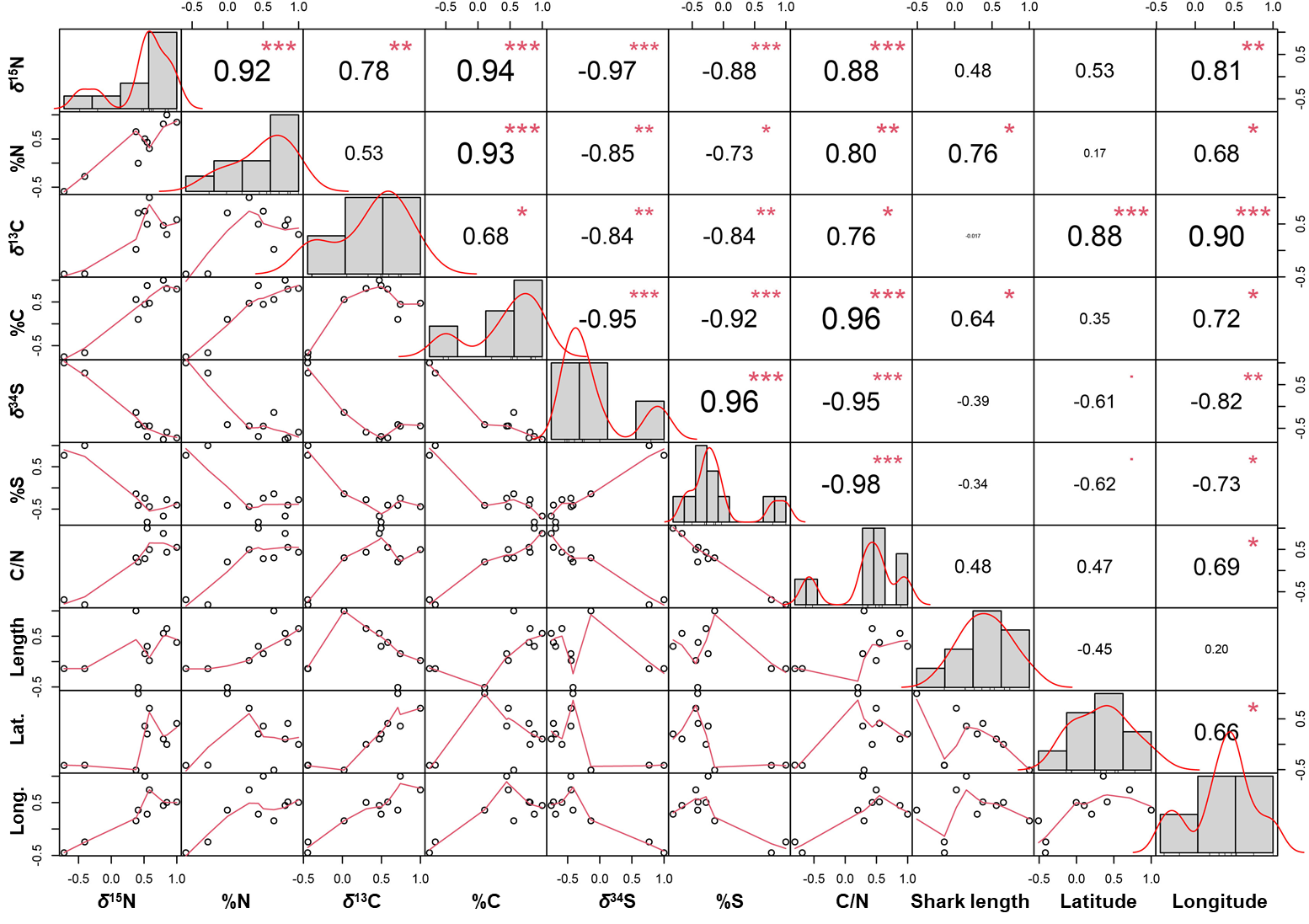


**Figure S1.** Correlation table of *E. granulosus*’ muscle tissues stable isotope values, elemental compositions and C/N ratios. Values were also compared with shark length (Length) and shark location: latitude (Lat.) and longitude (Long.). Coefficients in upper triangle corresponds to R values and red stars represents the level of significance: no star = not significant, * = p value < 0.05, ** = 0.05 <p value < 0.001 and *** = p values << 0.001.


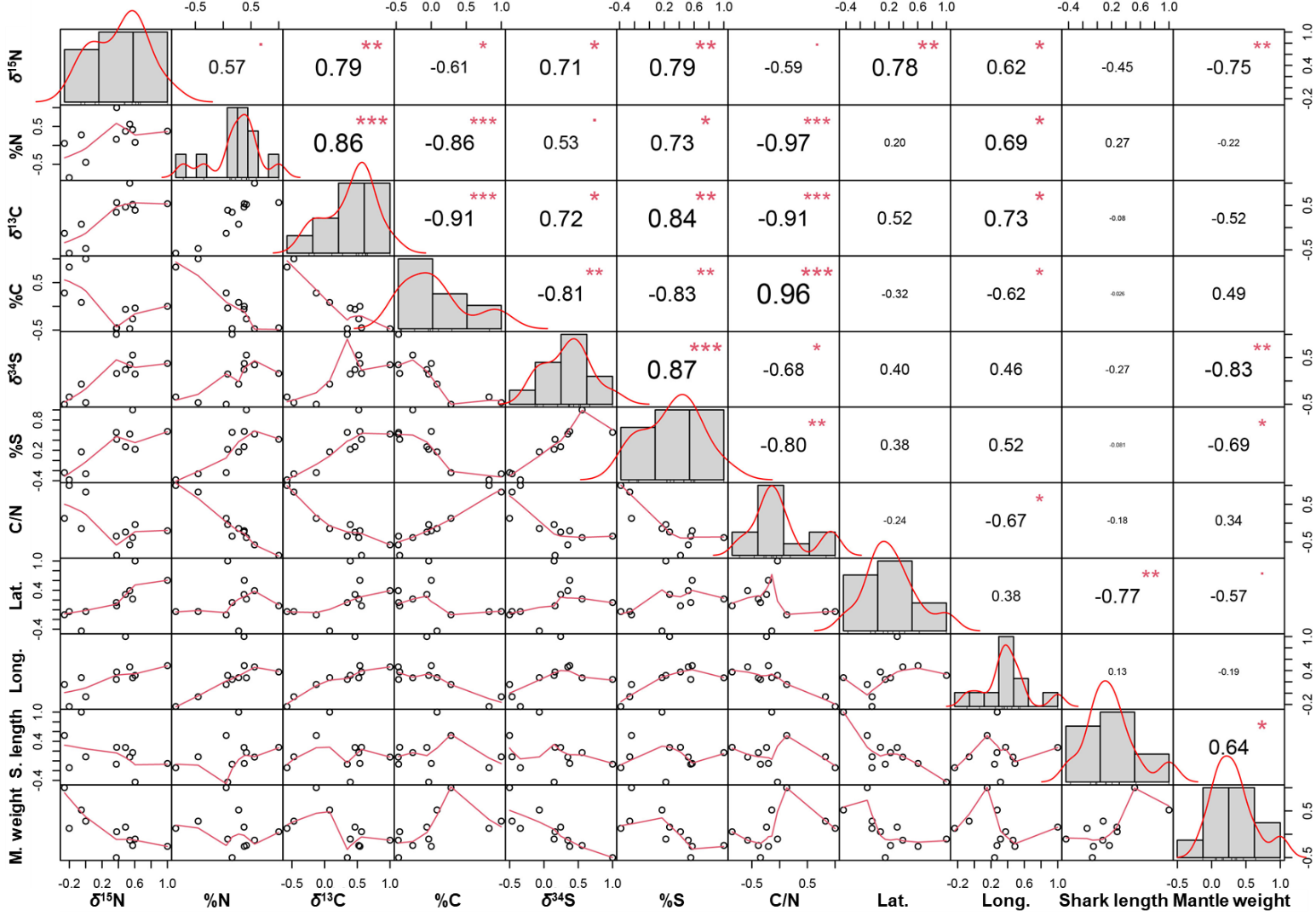


**Figure S2.** Correlation table of *A. squalicola*’s ‘protein tissues’ stable isotope values, elemental compositions and C/N ratios. Values were also compared with shark length (Length) and shark location: latitude (Lat.) and longitude (Long.) and barnacle weights (Mantle weight). Coefficients in upper triangle corresponds to R values and red stars represents the level of significance: no star = not significant, * = p value < 0.05, ** = 0.05 <p value < 0.001 and *** = p values << 0.001.


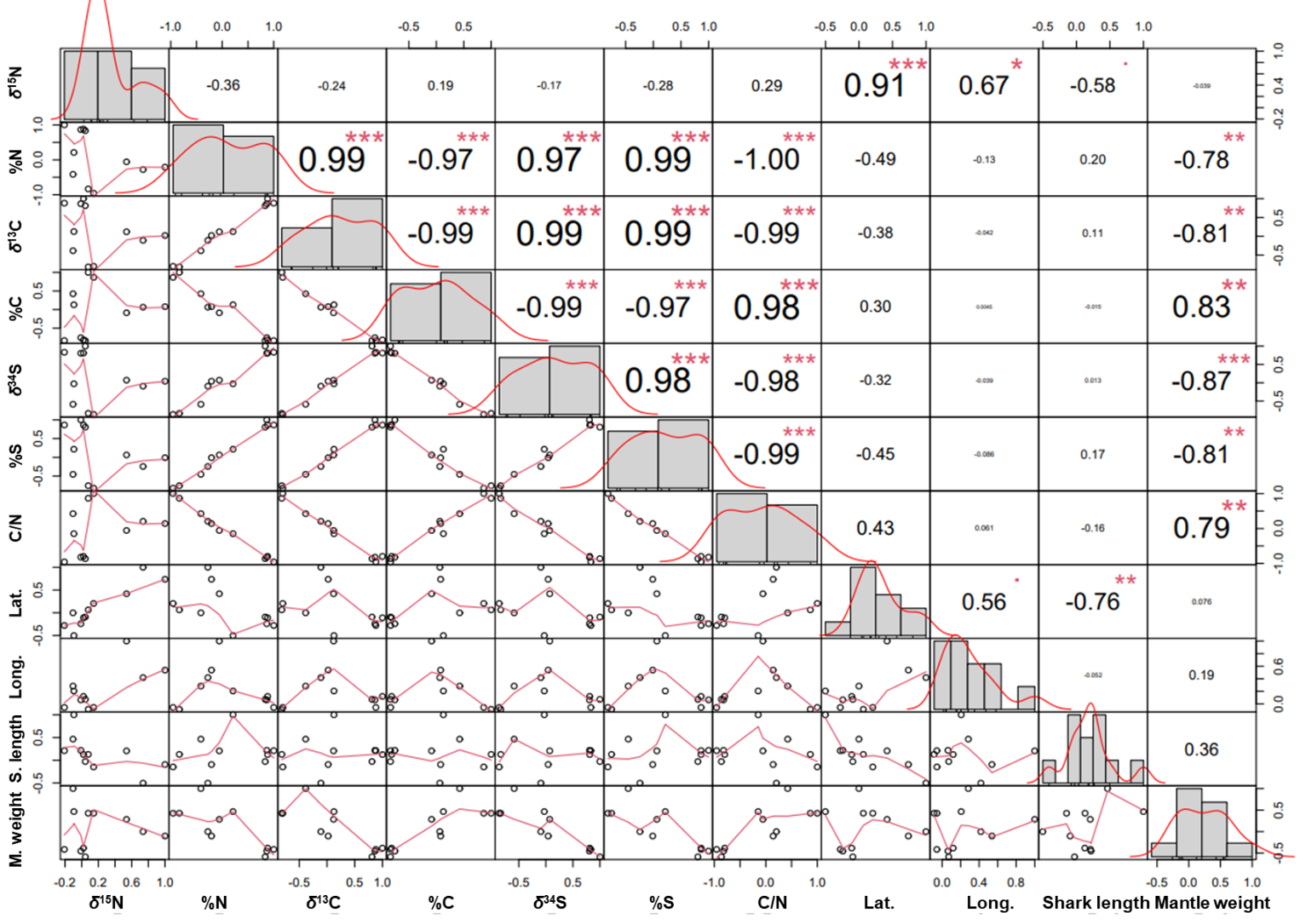


**Figure S3.** Correlation table of *A. squalicola*’s peduncle tissues stable isotope values, elemental compositions and C/N ratios. Values were also compared with shark length (Length) and shark location: latitude (Lat.) and longitude (Long.) and barnacle weights (Mantle weight). Coefficients in upper triangle corresponds to R values and red stars represents the level of significance: no star = not significant, * = p value < 0.05, ** = 0.05 <p value < 0.001 and *** = p values << 0.001.

*
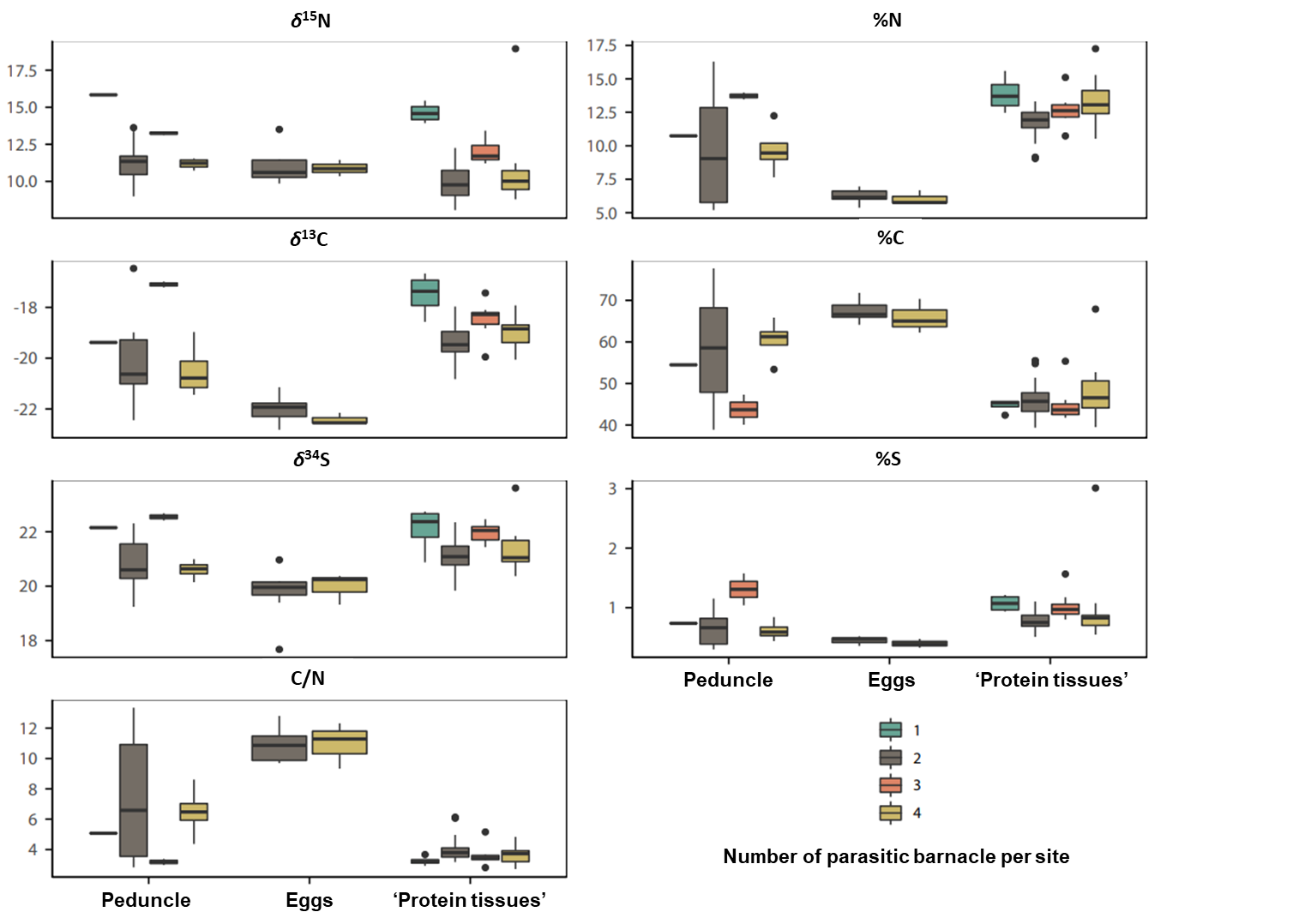
*

**Figure S4.** Boxplot highlighting the relationship between the number of *A. squalicola* per infection site on stable isotope values (*δ*^15^N, δ^13^C and δ^34^S), elemental compositions (%N, %C and %S) and the C/N ratio.

**Tables**

**Table S1.** Difference between host shark or parasitic barnacle tissues vs host shark ‘healthy’ muscle tissues for the different stable isotope values, elemental composition, and C/N ratio.


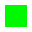

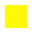


| **Host Shark** |  | **δ^15^N (‰)** | **%N** | **δ^13^C (‰)** | **%C** | **δ^34^S (‰)** | **%S** | **C/N** |
| --- | --- | --- | --- | --- | --- | --- | --- | --- |
| Δ‘unhealthy'-‘healthy’ muscle | Avg. | -1.0 | -3.3 | -0.7 | 0.7 | 0.7 | -0.1 | 0.8 |
|  | SD | 0.9 | 1.5 | 0.4 | 1.6 | 0.6 | 0.2 | 0.4 |
| ΔEye-‘healthy’ muscle | Avg. | -0.7 | 2.1 | 0.5 | n/a | -1.9 | -0.3 | n/a |
|  | SD | n/a | n/a | n/a | n/a | n/a | n/a | n/a |
| **Parasitic barnacle** |  |  |  |  |  |  |  |  |
| ΔPeduncle-‘healthy’ muscle | Avg. | -0.4 | -5.6 | -1.1 | 9.2 | 1.0 | -0.1 | 3.2 |
|  | SD | 1.4 | 3.0 | 1.8 | 11.2 | 1.0 | 0.3 | 3.1 |
| ΔEggs-‘healthy’ muscle | Avg. | -1.2 | -9.6 | -3.6 | 19.5 | 0.0 | -0.4 | 7.7 |
|  | SD | 1.1 | 1.5 | 0.5 | 4.4 | 0.4 | 0.1 | 1.1 |
| ΔProtein tissues-‘healthy’ muscle | Avg. | -1.8 | -3.7 | -0.4 | -0.9 | 1.2 | -0.1 | 0.8 |
|  | SD | 1.3 | 1.1 | 0.7 | 4.4 | 0.7 | 0.2 | 0.5 |

**Table S2.** ANOVA tests isotopic ratios. Results for eyes should be taken with caution, as based on only one value.

| Variable | Statistic | p value |  | Post hoc |
| --- | --- | --- | --- | --- |
| δ^15^N | F_109,8_ = 2.14 | 0.04 | * | Eye^ab^ = Inner mantle^a^ = MCP^ab^ = Rootlets^ab^ = Mantle^ab^ = Eggs^ab^ = ‘Unhealthy' shark muscle^ab^ = Peduncle^ab^ = ‘Healthy' shark muscle^b^ |
| %N | F_109,8_ = 24.64 | <2.2 10^-16^ | *** | Eggs^a^< Peduncle^b^< Mantle^c^ = Inner mantle^c^ = MCP^cd^ = Rootlets^c^ = ‘Unhealthy' shark muscle^cd^ = Eye^abc^ < ‘Healthy' shark muscle^d^ |
| δ^13^C | F_108,8_ = 14.16 | 6.3 10^-14^ | *** | Eggs^a^ < Peduncle^b^= ‘Unhealthy' shark muscle^bc^ = Inner mantle^bc^ = Eye^bc^ = Rootlets^bc^ = Mantle^bc^ < MCP^c^ = ‘Healthy' shark muscle^c^ |
| %C | F_109,8_ = 18.48 | <2.2 10^-16^ | *** | MCP^a^ = ‘Healthy' shark muscle ^a^ = Eye^a^ = Inner mantle^a^ = Rootlets^a^ = Mantle^a^ = ‘Unhealthy' shark muscle^a^ < Peduncle^b^ < Eggs^c^ |
| δ^34^S | F_109,8_ = 7.78 | 3.2 10^-8^ | *** | Eggs^a^ ≤ Eye^ab^ = ‘Healthy' shark muscle ^ab^ = ‘Unhealthy' shark muscle^ab^ < Peduncle^bc^ = Rootlets^bc^ = Inner mantle^bc^ = Mantle^bc^ = MCP^bc^ |
| %S | F_109,8_ = 3.98 | 3.6 10^-4^ | *** | Eggs^a^ ≤ Peduncle^ab^ = Inner mantle^ab^ ≤ Rootlets^b^ = MCP^b^ ≤ ‘Unhealthy' shark muscle^ab^ < Mantle^b^ = ‘Healthy' shark muscle ^b^ = Eye^ab^ |
| C:N | F_109,8_ = 32.43 | <2.2 10^-16^ | *** | ‘Healthy' shark muscle ^a^ = Eye^a^ = MCP^a^ = Rootlets^a^ = ‘Unhealthy' shark muscle^a^ = Inner mantle^a^ = Mantle^a^ < Peduncle^b^ < Eggs^c^ |
